# Biocontrol effects of *Bacillus velezensis* and *Bacillus subtilis* against strawberry root rot caused by *Neopestalotiopsis clavispora*

**DOI:** 10.3389/fmicb.2025.1683291

**Published:** 2025-11-17

**Authors:** Jin Ning, Tiao Ning, Lu Jin, Qiongfen Li, Yanfen Niu, Zebin Chen, Chengchou Han, Yilian Tang, Changjun Deng, Yingying Xie, Mingfang Zhao, Xingguo Cui, Jing Li

**Affiliations:** 1 Engineering Research Center for Urban Modern Agriculture of Higher Education in Yunnan Province, School of Agriculture and Life Sciences, Kunming University, Kunming, China; 2 Yunnan Ornamental Seedlings and Trees Industry Association, Kunming, China; 3 School of Mechanical and Electrical Engineering, Kunming University, Kunming, China; 4 Yunnan Hanzhe Technology Co., Ltd., Kunming, China; 5 Yulong County Jiuhe Xinxing Agricultural Development and Planting Co., Ltd., Lijiang, China; 6 Shangri-La Zangmei Agricultural Technology Co., Ltd., Diqing, China

**Keywords:** strawberry, strawberry root rot, *Bacillus velezensis*, *Bacillus subtilis*, antagonistic bacteria, colonization, biological control

## Abstract

Strawberry root rot, caused by *Neopestalotiopsis clavispora*, is an emerging disease that seriously threatens the sustainable development of the strawberry industry. To develop eco-friendly control strategies, three antagonistic bacterial strains were screened from healthy strawberry plants and rhizosphere soils. Based on morphological characteristics, physiological and biochemical identification, and 16S rDNA sequence analysis, the isolates QY-4 and QJ-3 were identified as *Bacillus velezensis*, while TT-3 was identified as *Bacillus subtilis*. The results indicated that the cell-free culture filtrates of QY-4, QJ-3, and TT-3 significantly inhibited the hyphal growth of *N. clavispora* by disrupting cell membrane integrity, with inhibition rates of 63.29, 69.4, and 73.57%, respectively. Volatile organic compounds produced by these strains, evaluated using the plate pair method, effectively inhibited hyphal growth through aerial diffusion with inhibition rates of 47.76, 44.99, and 32.44%. Broad-spectrum antagonistic activity against several phytopathogenic fungi, including *Colletotrichum acutatum*, *Alternaria alternata*, and *Botrytis cinerea*, was observed with inhibition rates ranging from 50.37 to 78.88%. Through the antibiotic marker method, the labeled strains were shown to translocate from roots to stems and leaves following root irrigation treatment, establishing stable colonization in both strawberry tissues and rhizosphere soils. The application of these antagonistic strains significantly alleviated root rot symptoms and markedly reduced the disease index, with values of 36.98, 42.19, and 27.92, corresponding to disease control efficiencies of 56.28, 50.12, and 67%, respectively. Additionally, significant enhancement of superoxide dismutase, peroxidase, and catalase was observed in leaves, indicating the induction of host resistance. These findings demonstrate the dual role of QY-4, QJ-3, and TT-3 as biocontrol agents, by combining antifungal activities with resistance induction, thus offering promising candidates for the sustainable management of strawberry root rot.

## 1 Introduction

Strawberry (*Fragaria* × *ananassa* Duch.) is a perennial herbaceous plant of the Rosaceae family native to South America and is globally valued for its succulent fruits with a sweet–tart flavor, tender texture, and distinctive aroma (Manganaris et al., 2014). However, its production is severely constrained by *Neopestalotiopsis clavispora*, the causal agent of strawberry root rot. This aggressive fungal pathogen induces characteristic symptoms such as reddish-brown leaf discoloration, root necrosis, and systemic wilting, ultimately resulting in substantial yield losses (30–70%) and quality deterioration (Basu et al., 2014; Ávila-Hernández et al., 2025). *N. clavispora* demonstrates a wide host range as a thermophilic and hygrophilic pathogen, infecting various economically important crops, including lingonberry (*Vaccinium vitis-idaea*) (Afrin et al., 2016), loquat (*Eriobotrya japonica*) (Giampieri et al., 2015), mango (*Mangifera indica*) (Hidrobo-Chavez et al., 2022), and blueberry (*Vaccinium corymbosum*) (Obregón et al., 2018). Current disease control strategies predominantly rely on the use of synthetic fungicides. However, prolonged application accelerates the development of fungicide-resistant *N. clavispora* strains and introduces environmental and health risks owing to pesticide residues (Mordue, 1985). Additionally, the lack of a protective epicarp in strawberry fruits renders them highly susceptible to chemical contamination, highlighting the urgency of developing eco-friendly alternatives (Wu et al., 2022).

Biological control using *Bacillus* spp. has emerged as a promising strategy because of its multiple advantages, including environmental compatibility, low risk of resistance development, enhanced stress tolerance, and ability to produce a wide range of antimicrobial metabolites (e.g., lipopeptides and polyketides) (Hokanson and Finn, 2000). Notably, *Bacillus velezensis* and *Bacillus subtilis* are among the most extensively studied biocontrol species. Numerous studies have reported that *B. velezensis* strains can synthesize antimicrobial lipopeptides, including surfactin, iturin, and fengycin, exhibiting significant antagonistic activity against various plant pathogenic fungi (Zhou et al., 2022; Keshmirshekan et al., 2024). Similarly, *B. subtilis* and its related strains are widely documented for their ability to effectively suppress soil-borne diseases through the production of antimicrobial compounds, induction of systemic resistance, and niche competition (Rafiee et al., 2022; Islam et al., 2012). These attributes make them excellent candidates for developing biocontrol agents. For instance, *Bacillus cereus* strain Bc-2 exhibited 79.48% inhibition of *N. clavispora* mycelial growth in vitro and achieved 57.85% reduction in disease severity under field conditions. It also significantly enhanced the activity of defense-related enzymes such as peroxidase (POD), superoxide dismutase (SOD), catalase (CAT), and phenylalanine ammonia-lyase (PAL) (Zhang et al., 2024). Despite these advances, research on *Bacillus*-mediated biocontrol of strawberry root rot remains limited, with only one *B. cereus* strain reported to date, underscoring the need to explore novel antagonistic candidates.

The isolation of bacteria from the soil and plant rhizosphere is a crucial approach for discovering beneficial microorganisms with biocontrol potential and plant growth-promoting properties. This niche is rich in microbial diversity and represents an ideal source for finding antagonistic strains capable of adapting to specific microenvironments and effectively colonizing plant roots (Morales-Cedeño et al., 2021).

To address these gaps, this study isolated three antagonistic bacterial strains with potent antifungal activity against *N. clavispora* from the healthy strawberry rhizosphere. Their potential was systematically evaluated in terms of (1) broad-spectrum antifungal activity against phytopathogens, (2) root colonization efficiency and disease suppression efficacy in plants, and (3) induction of defense-related enzymes [superoxide dismutase (SOD), peroxidase (POD), and catalase (CAT)] in strawberry leaves.

## 2 Materials and methods

### 2.1 Bacterial isolation

Strawberry plants and rhizosphere soils were collected from Huaning County, Hongta District, Yuxi City, Yunnan Province, China. A total of 6 healthy strawberry plants, along with their corresponding rhizosphere soil samples (approximately 15 g each), were collected as three biological replicates. Bacterial isolation was performed within 48 h to ensure sample freshness. Healthy strawberry plants were washed with sterile water, and the roots, stems, and leaves were cut into tissue blocks approximately 5 mm in length and 5 mm in width. Surface disinfection was performed using 75% ethanol (1 min), followed by 2% sodium hypochlorite (1 min), after which the samples were rinsed three times with sterile water. Leaf and stem tissues were soaked for 10 min. The root tissues were fully ground in sterile water, the resulting suspension was serially diluted (10^–2^–10^–8^), and 100 μL of each dilution was spread onto LB solid medium (Li et al., 2022). This step was repeated three times. Following 24 h of incubation at 28°C, single colonies with distinct morphologies were selected for subculturing, labeling, and preservation in 30% glycerol at −80°C.

For rhizosphere samples, 5 g of soil was transferred to a sterile tube containing 45 mL of sterile water. The mixture was shaken at 200 rpm for 30 min at 28°C and allowed to settle. A 1 mL aliquot of the supernatant was serially diluted (10^–2^–10^–8^), and 100 μL of the 10^–6^–10^–8^ dilution solution was plated on LB solid medium (Wagemans et al., 2022). After triplicate plating and incubation at 28°C for 24 h, morphologically distinct colonies were selected, subcultured, labeled, and stored in 30% glycerol at −80°C.

### 2.2 Screening of antagonistic bacteria

In this study, *N. clavispora* was selected as the target pathogen. Preliminary screening identified bacterial strains capable of significantly inhibiting the growth of *N. clavispora*. A 3 mm diameter fungal plug was placed at the center of a PDA plate, while bacterial plugs (3 mm in diameter) from different strains were placed 3 cm away from the fungal plug. Plates containing only the fungal plug served as controls (CK). Each group included three replicates and was incubated at 28 °C. Once the fungal colonies in the control group were fully developed, the colony radius was measured, and the inhibition rate was calculated using the following formula (Li et al., 2021).


Inhibitionrate(%)=(A-B)/A×100

where A represents the pathogen colony radius of the control group, and B represents the pathogen colony radius of the treatment group.

For secondary screening, the bacterial strains exhibiting the inhibition rates ≥ 50% were cultured in 10 mL LB liquid medium at 28 °C with shaking (180 rpm) for 18 h, and the cell density was adjusted to OD600 = 0.8. A 3 mm fungal plug was placed in the center of a PDA plate with sterile filter paper discs positioned 3 cm away from the fungal plug. Each disc was inoculated with 20 μL of bacterial culture suspension. Plates with 20 μL of LB medium served as blank controls. All treatments were performed in triplicate and incubated at 28 °C. Once the control colonies were fully developed, the diameter of fungal growth was measured, and the inhibition rates were calculated accordingly (Al-Mutar et al., 2023; Li et al., 2020).


Inhibitionrate(%)=(A-B)/A×100

where A is the pathogen colony diameter of a control group, and B is the pathogen colony diameter of the treatment group.

### 2.3 Identification of antagonistic bacteria

Antagonistic bacteria were cultured in LB medium at 38 °C for 3 days. Morphological identification was based on single-colony characteristics, whereas physiological and biochemical identification followed the protocols outlined in the standard bacterial identification manual (Dong and Cai, 2001). Single-colony plates of the antagonistic strains were submitted to Shanghai Shenggong Bioengineering Co., Ltd. (China) for 16S rDNA sequencing. Homology analysis was conducted using the BLAST program in the NCBI database, and a phylogenetic tree was constructed using the neighbor-joining method in MEGA 7.0.

### 2.4 Inhibitory effects of cell-free culture filtrates and volatile organic compounds from antagonistic bacteria on *N. clavispora*

#### 2.4.1 Preparation of antagonistic bacterial seed cultures

Antagonistic bacteria were purified using the streak plate method and cultured at 28 °C for 24 h. A single colony was transferred to 100 mL of LB liquid medium and incubated with shaking at 180 rpm for 18 h at 28 °C.

#### 2.4.2 Inhibitory effect of aseptic fermentation filtrate on mycelia growth of *N. clavispora*

The seed solution of the antagonistic bacteria was inoculated into LB medium at a concentration of 5% (v/v) and cultured with shaking at 28 °C (180 rpm) for 5 days to obtain the fermentation broth. The broth was centrifuged at 10,000 rpm for 10 min, and the supernatant was filtered three times through a 0.22 μm microporous membrane to obtain aseptic fermentation filtrate. A PDA medium containing sterile fermentation filtrate (volume ratio of filtrate to PDA medium: 1:1) was prepared. The pathogen cake was inoculated at the center of the PDA plate, whereas the control plate was inoculated with an equal volume of sterile water. Each treatment was repeated three times, and all plates were incubated at 28 °C until the control colony fully overgrew the plate (Li Y. et al., 2023).

#### 2.4.3 Effects of different concentrations of aseptic fermentation filtrate on membrane permeability of *N. clavispora*

Spores were produced by culturing the pathogen on PDA at 28°C. After collection, the spores were washed with 5 mL of sterile water and diluted to 1 × 10^8^ spores/mL. A total of 1 mL of the spore suspension was inoculated into 50 mL of PDB medium. After 7 days of shaking at 28°C (180 rpm), different concentrations (0.5, 5, and 50%) of aseptic fermentation filtrate were added. Culture medium without filtrate served as the control. Each treatment was repeated three times. After 24 h of shaking, 5 mL of culture solution was centrifuged (1,000 rpm, 10 min), and the conductivity of the supernatant was measured (Liu et al., 2024).

#### 2.4.4 Inhibitory effect of volatile organic compounds on mycelial growth of *N. clavispora*

The inhibitory effects of VOCs produced by antagonistic bacteria were evaluated using the plate pair method (Yamamoto et al., 2015). LB plates were coated with the antagonistic strain and cultured for 24 h. The PDA plate was inoculated with a pathogenic culture and sealed with film. A sealed double plate without antagonistic bacteria served as the control. Each treatment was repeated three times, and incubation was conducted at 28°C. Upon full growth of the control colony, the colony diameter was measured using the cross-crossing method, and the antibacterial rate was calculated.

### 2.5 Determination of the inhibitory spectra of antagonistic bacteria

The inhibitory spectra of the selected antagonistic strains were assessed using a dual-culture plate assay (Zhou et al., 2022). Seven pathogenic fungal strains were used as targets (Table 1), and those exhibiting antagonistic activity during preliminary screening were selected. PDA plates were inoculated with each target fungus alone for comparison. Each treatment was performed in triplicate and incubated at 28°C under constant temperature. When the control group showed complete fungal overgrowth, the colony diameter of the pathogenic fungi was measured using the cross-streak method, and the inhibition rates were calculated.

**TABLE 1:** Information on pathogenic fungi tested.

Pathogen species	Susceptible host plant
*C. acutatum*	Strawberry
*C. adiposa*	Water chestnut
*A. alternata*	Konjac
*B. dothidea*	Walnut
*F. equiseti*	Chrysanthemum
*F. solani*	Flue-cured tobacco
*B. cinerea*	Cigar tobacco

### 2.6 Colonization ability of antagonistic bacterial strains in strawberry plants and rhizosphere soil

An antibiotic marker method was used to track the colonization of antagonistic bacteria (Wu et al., 2022). The bacterial strains were cultured in LB broth for 3 d and then plated on LB agar containing 5 μg/mL rifampicin, followed by incubation at 28°C for 24 h. Colonies exhibiting morphological characteristics identical to those of the original strain were selected and transferred to LB agar containing 10 μg/mL rifampicin. This procedure was repeated with progressively increasing rifampicin concentrations until stable mutants capable of growing on 100 μg/mL rifampicin-containing LB agar were obtained. The marked strains were then cultured in LB broth supplemented with 100 μg/mL rifampicin for 3 d, diluted with sterile water to a final concentration of 3 × 10^8^ CFU/mL, and used for inoculation. Strawberry seedlings were subjected to root wounding, and 100 mL of bacterial suspension was applied to each plant by root drenching (10 plants per group, with three replicates). At 1, 3, 5, 7, and 14 days post-inoculation, five randomly selected plants per group were sampled for bacterial isolation from roots, stems, leaves, and rhizosphere soil. For plant tissue analysis, 1 g of composite samples (roots/stems/leaves) was surface-sterilized, homogenized, and serially diluted (10^2^–10^5^). Aliquots (100 μL) were plated on rifampicin-supplemented (100 μg/mL) LB agar and incubated at 28°C for 48 h. Rhizosphere soil samples (1 g) were suspended in 10 mL of 0.85% NaCl, shaken at 160 rpm for 30 min, serially diluted, and plated. All experiments were performed in triplicate. Colony counts were recorded and used to calculate the total viable cells of the marked strains (CFU/g tissue or soil).

### 2.7 Biocontrol efficacy of antagonistic bacteria against strawberry root rot

Experimental Soil: Sterilized substrate potting mix (121°C, 20 min), cooled before use.

Plant Material: Uniformly healthy “Qianmei No.1” strawberry (*Fragaria × ananassa*) seedlings were transplanted (one plant/pot) and acclimatized for 15 days before experiments.

Inoculation Protocol: Root wounding: Two fibrous roots were severed per plant with a scalpel.

Bacterial treatment: 1 × 10^8^ CFU/mL antagonistic strain fermentation broth (root drench).

Pathogen challenge: 1 × 10^8^ spores/mL *N. clavispora* suspension (applied 48 h post-bacterial treatment where applicable).

Experimental Design (eight groups, 10 plants/group, and three replicates):

QY-4 antagonistic strain only;

QJ-3 antagonistic strain only;

TT-3 antagonistic strain only;

*N. clavispora* + QY-4 (pathogen → 48 h → bacterium);

*N. clavispora* + QJ-3 (pathogen → 48 h → bacterium);

*N. clavispora* + TT-3 (pathogen → 48 h → bacterium);

*N. clavispora* only (positive control);

Sterile water (negative control).

Disease Assessment: Disease symptoms were monitored beginning 10 d post-inoculation. Final disease severity was evaluated at 30 days using Vestberg’s 6-grade scale (Vestberg et al., 2004): Grade 0: healthy plant; Grade 1: ≤ 30% root infection (healthy leaves); Grade 2: 30–60% root infection (healthy leaves); Grade 3: 60–80% root infection (leaf yellowing); Grade 4: > 80% root infection (leaf wilting); Grade 5: plant death


Diseaseseverityindex(DSI)=100×Σ(diseasegrade×

plantnumber)/(totalplants×highestgrade)

Biocontrolefficacy=100%×(controlDSI-treatmentDSI)/

control⁢DSI

### 2.8 Effects of root rot and antagonistic bacteria on strawberry defense enzymes sampling

Leaves were randomly sampled from each experimental group at 0, 1, 3, 5, and 7 days after treatment. The activities of superoxide dismutase (SOD), peroxidase (POD), and catalase (CAT) in leaf samples from all treatment groups were measured following the manufacturer’s instructions using commercial assay kits (Sangon Biotech Co., Ltd., Shanghai, China) (Meng et al., 2024).

### 2.9 Data analysis

Statistical analysis was conducted using SPSS 26.0 software. One-way analysis of variance (ANOVA) was performed to evaluate the intergroup differences among groups, followed by Duncan’s new multiple range test for *post hoc* comparisons. A significance threshold of *P* < 0.05 was applied to all statistical assessments.

## 3 Results

### 3.1 Isolation, purification, and screening of antagonistic bacteria

**FIGURE 1:**
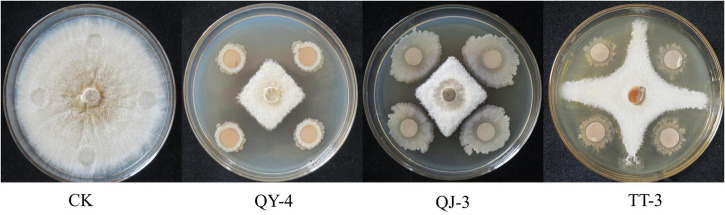
Antagonistic effects of the three antagonistic bacteria on *N. clavispora.*

In total, 113 bacterial strains were isolated from healthy strawberry plants and rhizosphere soils. Preliminary screening identified 20 strains that exhibited strong inhibitory effects on the mycelial growth of *N. clavispora* (Supplementary Table 1). Secondary screening further confirmed that the fermentation broths of three strains, designated QY-4, QJ-3, and TT-3, exhibited significant antagonistic activity against *N. clavispora* (Figure 1), with inhibition rates of 64.45, 61.01, and 60.60%, respectively (Table 2).

**TABLE 2:** Antifungal activities of 20 isolated bacteria on mycelial growth of *N. clavispora.*

Strains	Inhibition rate (%)	Strains	Inhibition rate (%)
TJ-5	50.64 ± 0.57	QY-4	64.45 ± 0.53
TJ-9	22.97 ± 1.54	QJ-3	61.01 ± 1.08
TJ-12	36.54 ± 0.91	QG-6	49.77 ± 0.47
TG-8	50.23 ± 1.19	QG-7	59.68 ± 0.13
TT-2	58.27 ± 0.66	QT-1	59.83 ± 0.4
TT-3	60.6 ± 0.85	QT-2	59.67 ± 1.14
TT-13	51.42 ± 1.04	QT-3	57.47 ± 0.27
TT-15	57.91 ± 0.46	QT-12	32.72 ± 0.12
TT-19	43.48 ± 0.98	QT-14	18.49 ± 0.51
QY-1	51.36 ± 0.69	QT-15	20.13 ± 0.63

Data are presented as mean ± standard deviation.

### 3.2 Morphological, physiological, biochemical characteristics, and molecular identification of antagonistic bacteria

**FIGURE 2:**
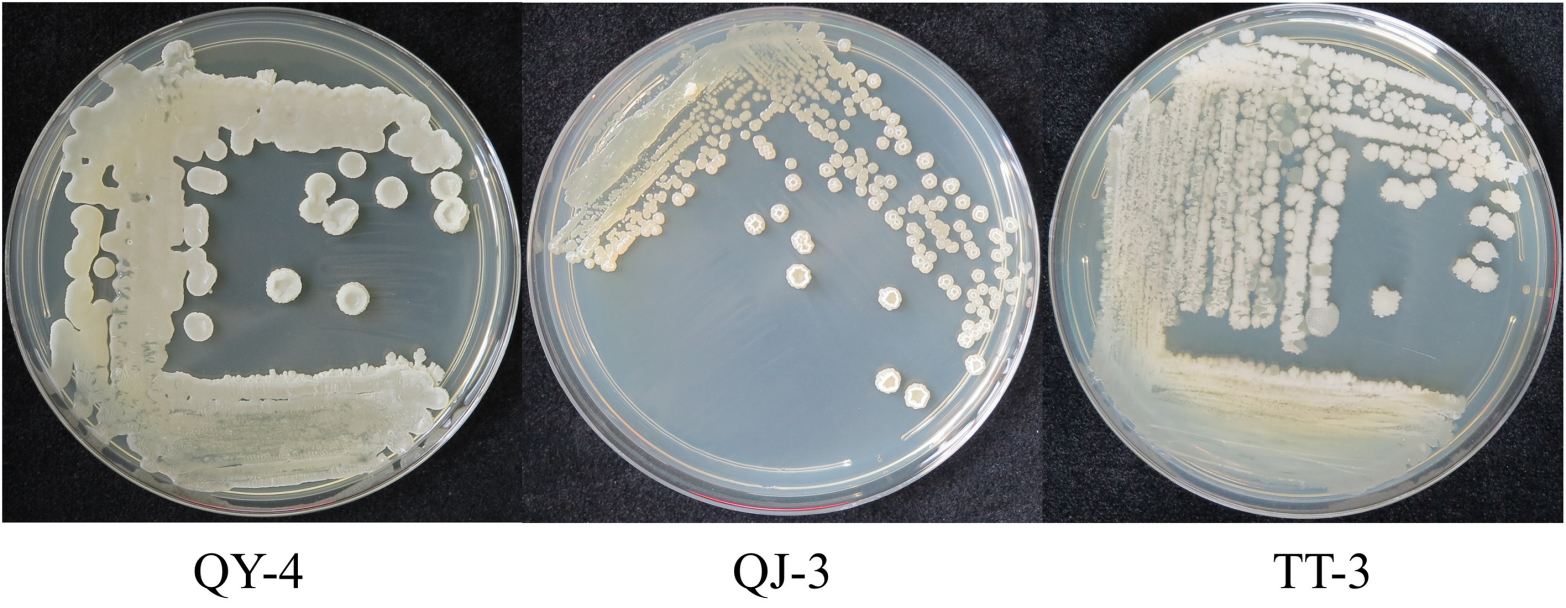
Morphological characteristics of the three antagonistic bacterial strains.

Colonies of strains QY-4 and QJ-3 appeared white with circular margins, smooth and opaque surfaces, and slightly concave raised centers. In contrast, strain TT-3 exhibited irregular margins and a rough, opaque surface (Figure 2). Physiological and biochemical analyses (Supplementary Table 2) indicated that all three antagonistic strains were Gram-positive, spore-forming bacteria capable of utilizing carbon sources such as glucose and sucrose. Enzymatic activities, including amylase, gelatinase, and catalase production as well as nitrogen gas release via denitrification, were detected. Based on these characteristics, the strains were preliminarily assigned to the genus *Bacillus*.

**FIGURE 3:**
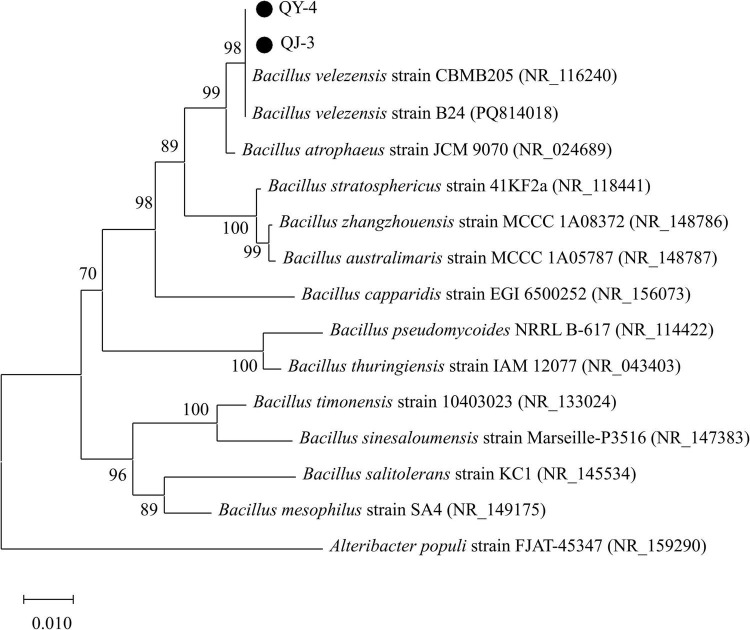
NJ phylogenetic tree of antagonistic strains QY-4 and QJ-3 based on 16S rDNA sequences.

**FIGURE 4:**
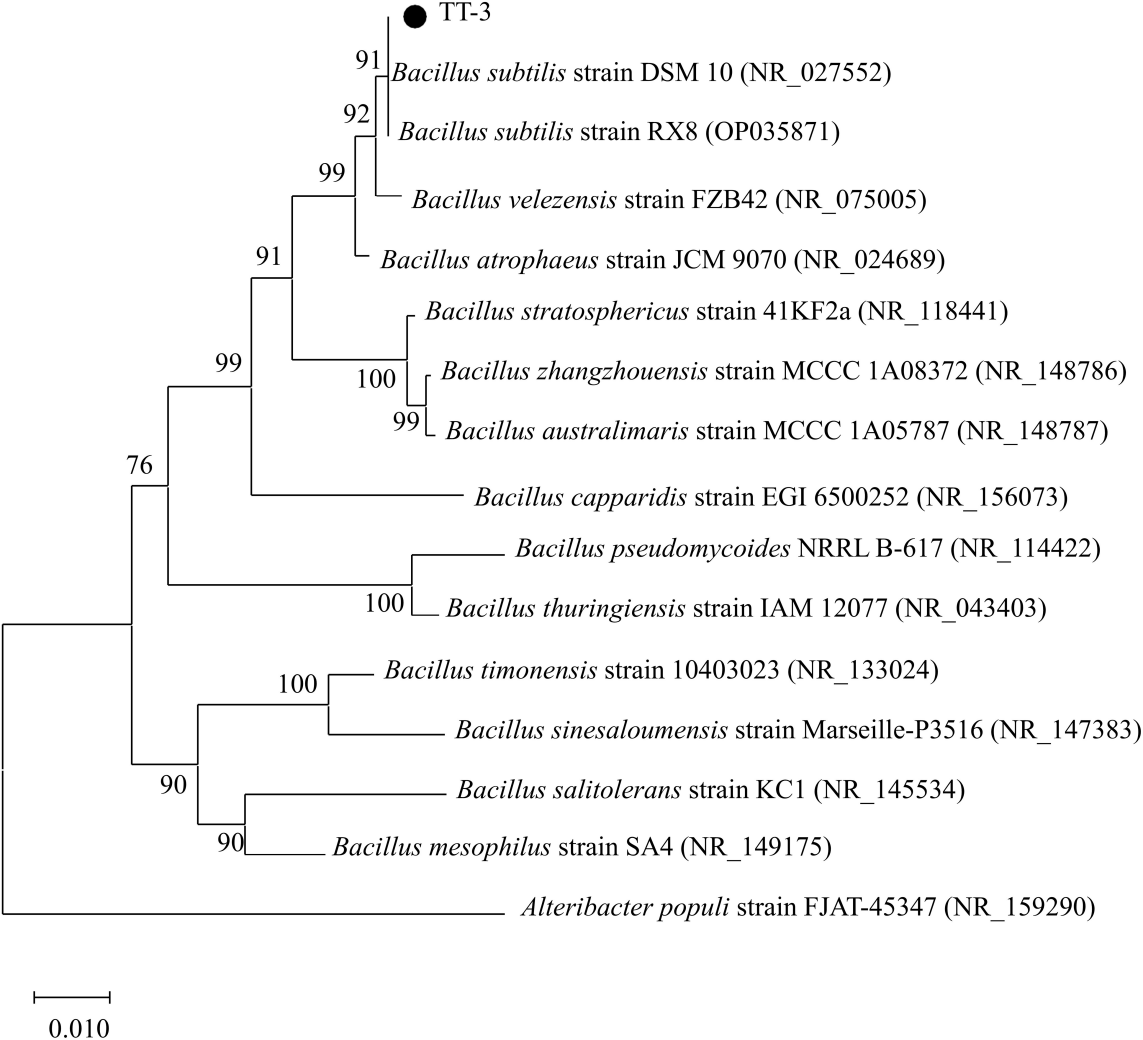
NJ phylogenetic tree of the antagonistic strain TT-3 based on 16S rDNA sequences.

Phylogenetic analysis of the 16S rDNA gene sequences revealed that strains QY-4 and QJ-3 shared the highest sequence similarity with *Bacillus velezensis* CBMB205 (NR_116240) and *Bacillus velezensis* B24 (PQ814018), supporting their classification within the *Bacillus velezensis* clade (Figure 3). Strain TT-3 exhibited the closest genetic relationship to *Bacillus subtilis* DSM10 (NR_027552) and *Bacillus subtilis* RX8 (OP035871), indicating its affiliation with the *Bacillus subtilis* group (Figure 4). The integration of morphological, physiological, and biochemical features with molecular data confirmed the identification of QY-4 and QJ-3 as *Bacillus velezensis*, whereas TT-3 was identified as *Bacillus subtilis*.

### 3.3 Effects of cell-free culture filtrates and volatile antimicrobial compounds from antagonistic bacteria on mycelial growth of *N. clavispora*

**FIGURE 5:**
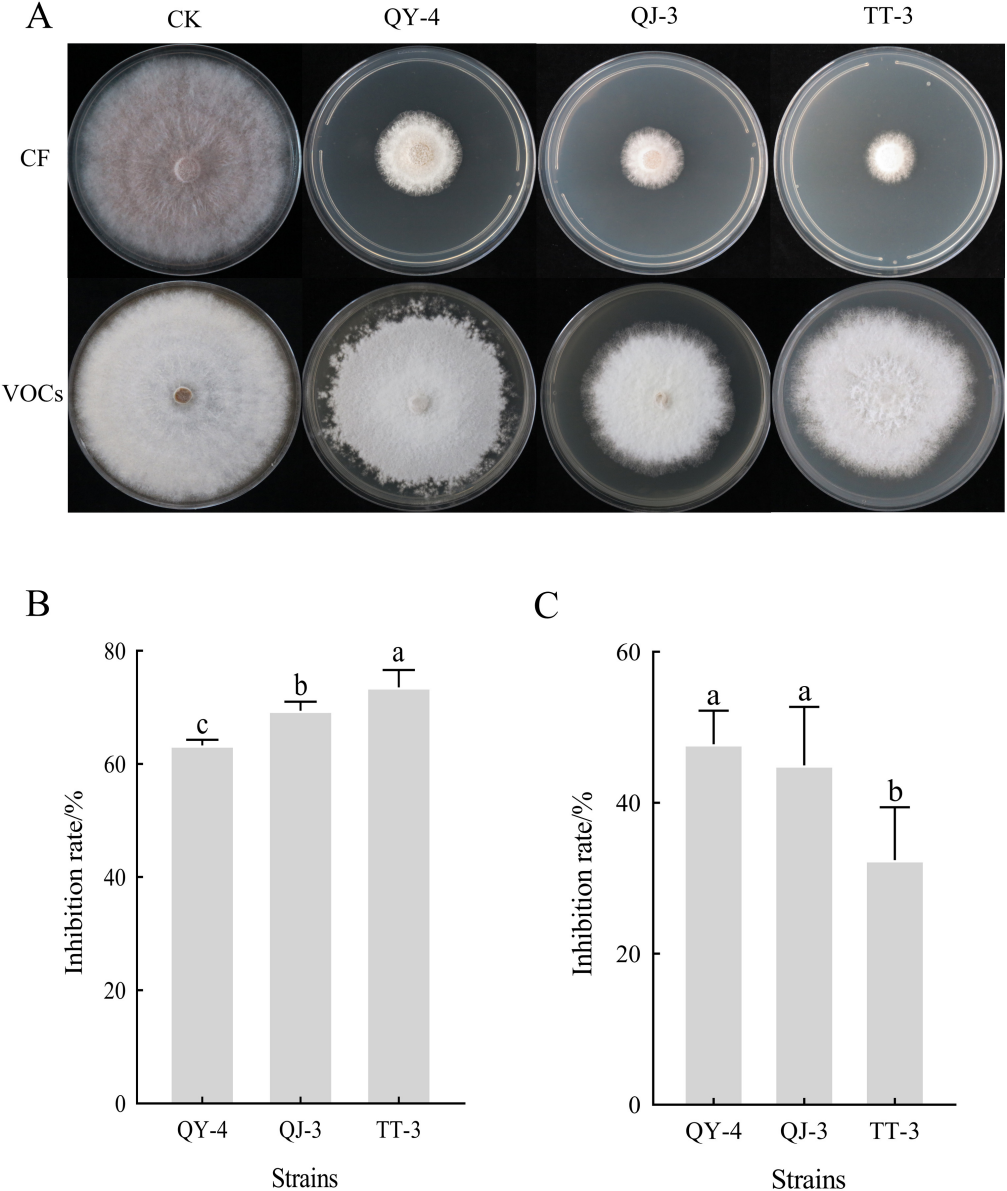
Antagonism of the three antagonistic bacterial strains against *N. clavispora*. **(A)** Antifungal Efficacy of cell-free culture filtrates and volatile organic compounds from three antagonistic bacterial strains against *N. clavispora*. **(B)** Inhibition rate of cell-free culture Filtrates of the three antagonistic bacteria on *N. clavispora*. **(C)** Inhibition rate of volatile organic compounds of the three antagonistic bacteria on *N. clavispora*. CF, cell-free culture filtrates; VOCs, volatile antimicrobial compounds; CK (control), *N. clavispora* grown on PDA plates without treatment. X-axis: antagonistic strains (QY-4, QJ-3,and TT-3); Y-axis: inhibition rate. Error bars: SD (*n* = 3). Different letters indicate significant differences (*p* < 0.05).

The cell-free culture filtrates (CF) of strains QY-4, QJ-3, and TT-3 exhibited strong inhibitory activity against mycelial growth of *N. clavispora* (Figure 5A), with inhibition rates of 63.29, 69.40, and 73.57%, respectively (Figure 5B). Alterations in cell membrane permeability disrupt intracellular homeostasis, resulting in the leakage of ions, nucleic acids, and other cytoplasmic components, thereby increasing extracellular electrical conductivity (Chen et al., 2020). To assess the effect of CF on membrane integrity, three concentrations (0.5, 5, and 50%) were tested. A concentration-dependent increase in the electrical conductivity of the culture medium was observed (Supplementary Figure 1), indicating that the non-volatile antimicrobial metabolites produced by these strains potentially compromised membrane integrity, triggered cytoplasmic efflux, and ultimately destabilized pathogen structures.

In contrast, the volatile organic compounds (VOCs) released by strains QY-4, QJ-3, and TT-3 exhibited comparatively weaker suppression of *N. clavispora* mycelial growth, with inhibition rates of 47.76, 44.99, and 32.44%, respectively (Figure 5C).

### 3.4 Determination of the antimicrobial spectrum of antagonistic bacteria

**FIGURE 6:**
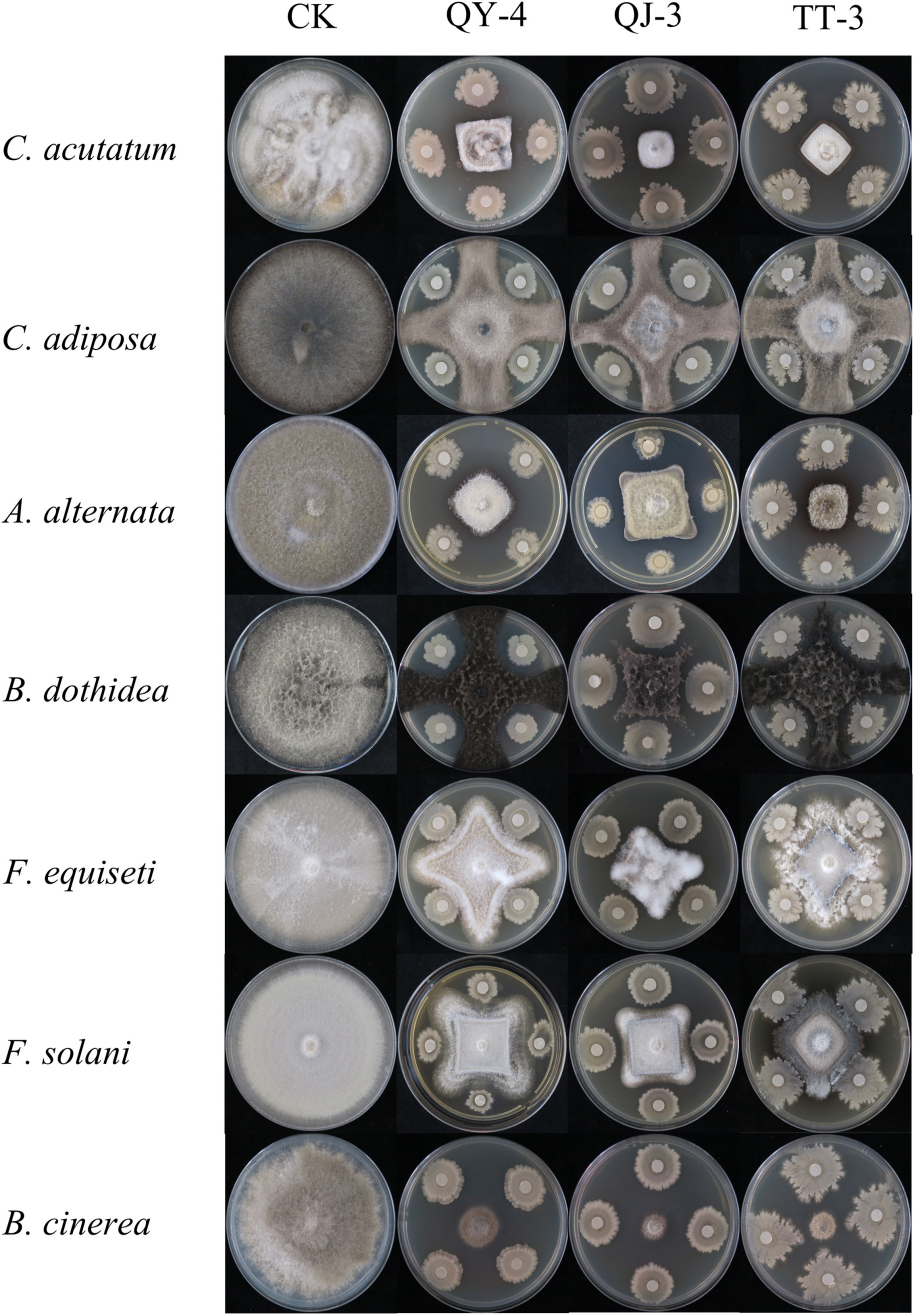
Antagonistic effect of three antagonistic bacteria on seven pathogenic fungi.

In addition to their potent antagonistic activity against the target pathogen *N. clavispora*, the three antagonistic strains exhibited broad-spectrum inhibitory effects against seven additional phytopathogenic fungi (Figure 6). As presented in Table 3, strain QY-4 achieved significantly higher inhibition rates against *Colletotrichum acutatum*, *Alternaria alternata*, and *Botrytis cinerea* (72.59, 72.26, and 74.48%, respectively) than the other four pathogens. Strain QJ-3 exerted the most pronounced inhibitory effects on *C. acutatum* and *B. cinerea*, with inhibition rates of 76.54 and 78.88%, respectively. This was followed by the moderate suppression of *A. alternata* and *Botryosphaeria dothidea* (71.09 and 70.12%, respectively). Strain TT-3 displayed the highest inhibition against *B. cinerea* (76.09%), with secondary activity observed against *A. alternata* and *C. acutatum* (71.22 and 69.68%, respectively).

**TABLE 3:** Antifungal activity of three antagonistic bacteria against seven pathogenic species.

Patho-genic fungi	Inhibition rate (%)		
	QY-4	QJ-3	TT-3
*F. oxysporum*	72.59 ± 3.7a	76.54 ± 0.69a	69.68 ± 1.73b
*C. adiposa*	50.37 ± 3.5c	57.68 ± 2.51e	54.19 ± 0.69d
*A. alternata*	72.26 ± 3.11a	71.09 ± 4.57b	71.22 ± 3.76b
*B. dothidea*	65.47 ± 10.53b	70.12 ± 2.87b	62.81 ± 3.78c
*F. equiseti*	55.38 ± 6.98c	63.8 ± 1.54c	50.54 ± 1.71e
*F. solani*	53.81 ± 7.95c	60.62 ± 4.14d	55.04 ± 2.7d
*B. cinerea*	74.48 ± 2.87a	78.88 ± 1.79a	76.09 ± 8.66a

Data are presented as mean ± standard deviation. Values with different letters in the same column are significantly different (*p* < 0.05).

### 3.5 Colonization capacity of three antagonistic bacterial strains in strawberry plants and rhizosphere soil

**FIGURE 7:**
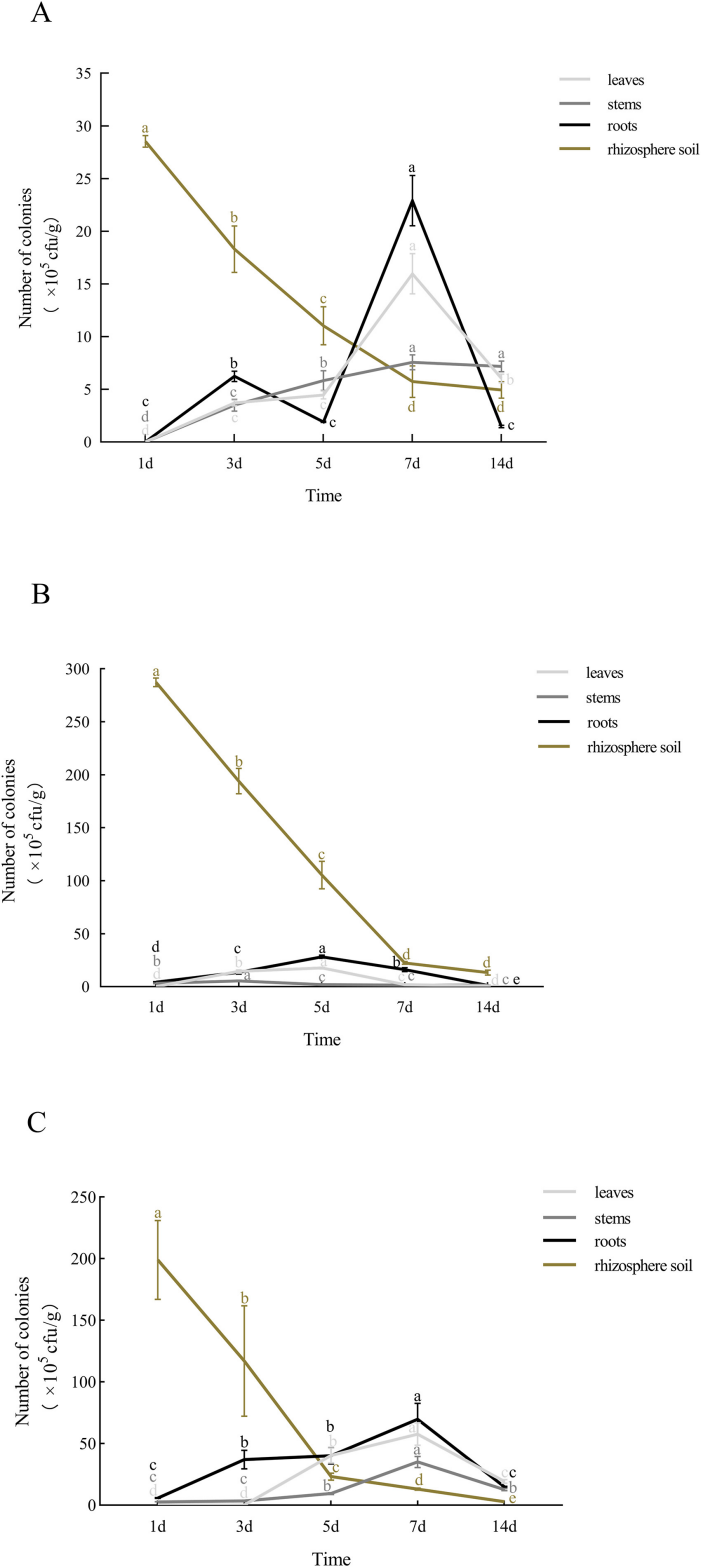
Colonization patterns of the three labeled antagonistic bacteria in strawberry tissues and rhizosphere soil. **(A)** Population dynamics of QY-4 stain in strawberry plants and rhizosphere soil. **(B)** Population dynamics of QJ-3 stain in strawberry plants and rhizosphere soil. **(C)** Population dynamics of TT-3 stain in strawberry plants and rhizosphere soil. The colonization dynamics of the three antagonistic bacteria in strawberry tissues and rhizosphere soil were revealed by the curves. X-axis: time; Y-axis: bacterial count (CFU/g). Error bars: SD (*n* = 3). Letters indicate significant temporal differences (*p* < 0.05).

Colonization with antagonistic bacteria is essential for effective biocontrol. Following root irrigation with the labeled strains of the three antagonistic bacteria, the strains were re-isolated from strawberry leaves, stems, roots, and rhizosphere soil, confirming their capacity to colonize both plant tissues and the rhizosphere and to translocate upward from roots to stems and leaves. The colonization dynamics of the labeled strains were as follows:

Strain QY-4 was detected in leaves, stems, and roots by day 3, with colonization peaking on day 7 (leaves: 16 × 105 CFU/g; stems: 7.6 × 105 CFU/g; roots: 22.9 × 105 CFU/g). Colonization levels gradually declined but remained relatively high thereafter. In the rhizosphere, the density decreased from 28 × 105 CFU/g to 5.7 × 105 CFU/g and subsequently stabilized (Figure 7A).

Strain QJ-3 exhibited the highest colonization density in the rhizosphere, followed by roots and leaves, with minimal colonization observed in stems. Rhizosphere colonization declined from 287.3 × 105 CFU/g to 22.4 × 105 CFU/g before stabilizing. The colonization in leaves and roots peaked on day 5 (17.7 × 105 CFU/g and 28.2 × 105 CFU/g, respectively), while the stem colonization peaked on day 3 (5.5 × 105 CFU/g) (Figure 7B).

For strain TT-3, the rhizosphere colonization decreased from 199 × 105 CFU/g to 2.9 × 105 CFU/g and stabilized. From days 1 to 7 post-inoculation, the colonization levels in leaves, stems, and roots increased from 0, 5.8 × 105, and 5.8 × 105 to 57.7 × 105, 35 × 105, and 69.7 × 105 CFU/g, respectively (Figure 7C).

All three strains exhibited a rapid initial decline in rhizosphere colonization, followed by stabilization, while maintaining persistent colonization of the plant vascular system and rhizosphere environment. Notably, strains QY-4 and TT-3 demonstrated superior endophytic colonization capacity within plant tissues.

### 3.6 Pot experiment evaluating the disease control efficacy of three antagonistic bacterial strains

**FIGURE 8:**
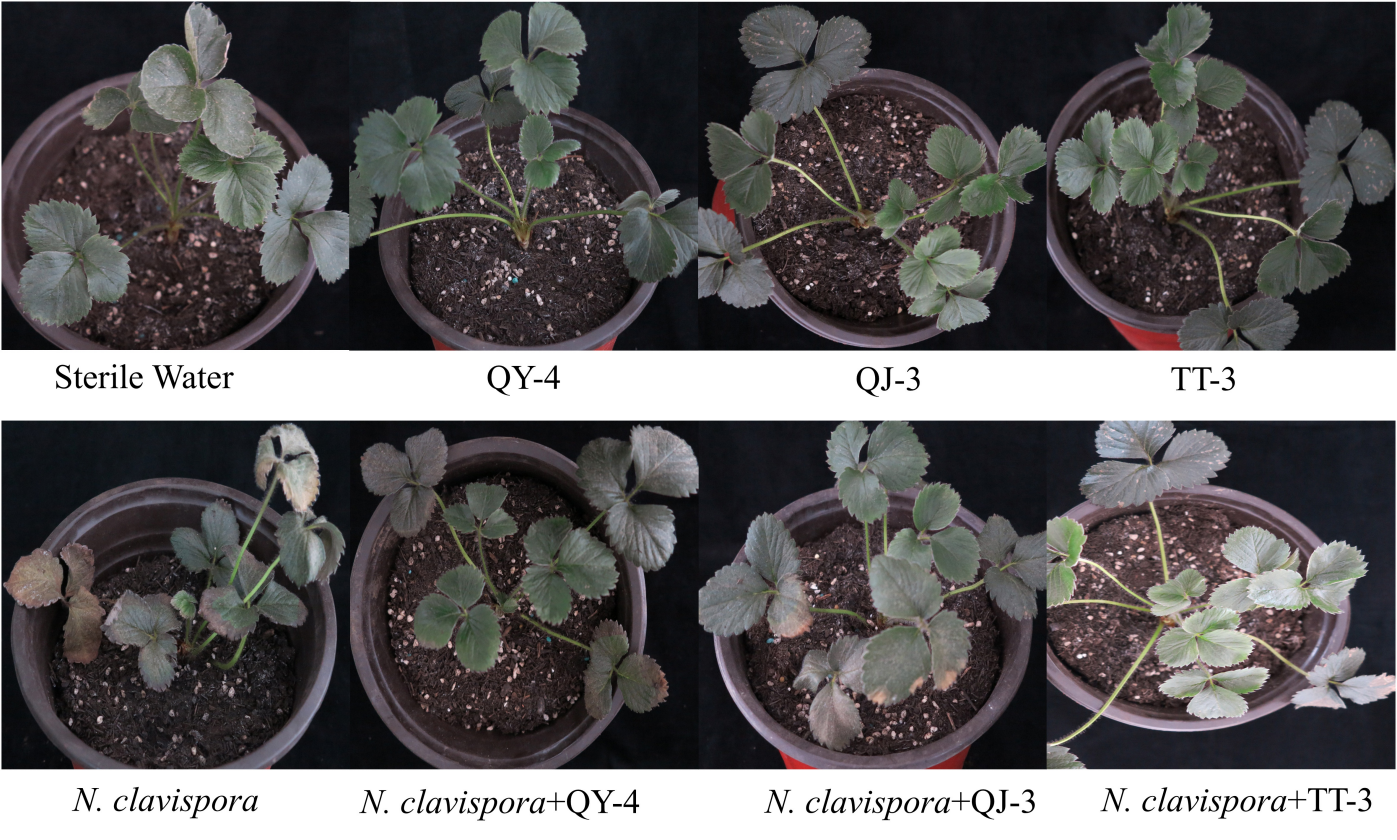
Strawberry indoor potted plant appearance.

To evaluate the disease control efficacy of antagonistic bacterial strains, a pot experiment was conducted to assess their potential in managing strawberry root rot caused by *N. clavispora*. As shown in Figure 8 and Table 4, strawberry plants inoculated solely with strains QY-4, QJ-3, or TT-3 maintained healthy growth at 30 d post-inoculation, confirming the absence of phytotoxic effects associated with the bacterial treatments. In contrast, plants inoculated with *N. clavispora* alone exhibited progressively more severe root rot symptoms, with a disease severity index (DSI) of 84.58 by 30 d. Co-inoculation with antagonistic strains markedly mitigated disease progression. Specifically, *N. clavispora* + QY-4 treatment reduced the DSI to 36.98, corresponding to a control efficacy of 56.28%. Similarly, co-inoculation with QJ-3 lowered DSI to 42.19 (50.12% efficacy). Notably, the combination of *N. clavispora* and TT-3 exhibited the most substantial suppression, reducing DSI to 27.92, with a control efficacy of 67.00%. These findings demonstrated that all three strains effectively reduced disease incidence and severity, with strain TT-3 providing the highest protective effect against strawberry root rot caused by *N. clavispora*.

**TABLE 4:** Indoor pot experiment results.

Treatments	Disease severity index	Control efficacy (%)
QY-4	0	–
QJ-3	0	–
TT-3	0	–
*N. clavispora*+QY-4	36.98	56.28
*N. clavispora* +QJ-3	42.19	50.12
*N. clavispora* +TT-3	27.92	67
*N. clavispora*	84.58	–
Sterile Water	0	–

### 3.7 Effects of the pathogen causing strawberry root rot of *N. clavispora* and antagonistic bacteria on defense enzyme activities in strawberry plants

**FIGURE 9:**
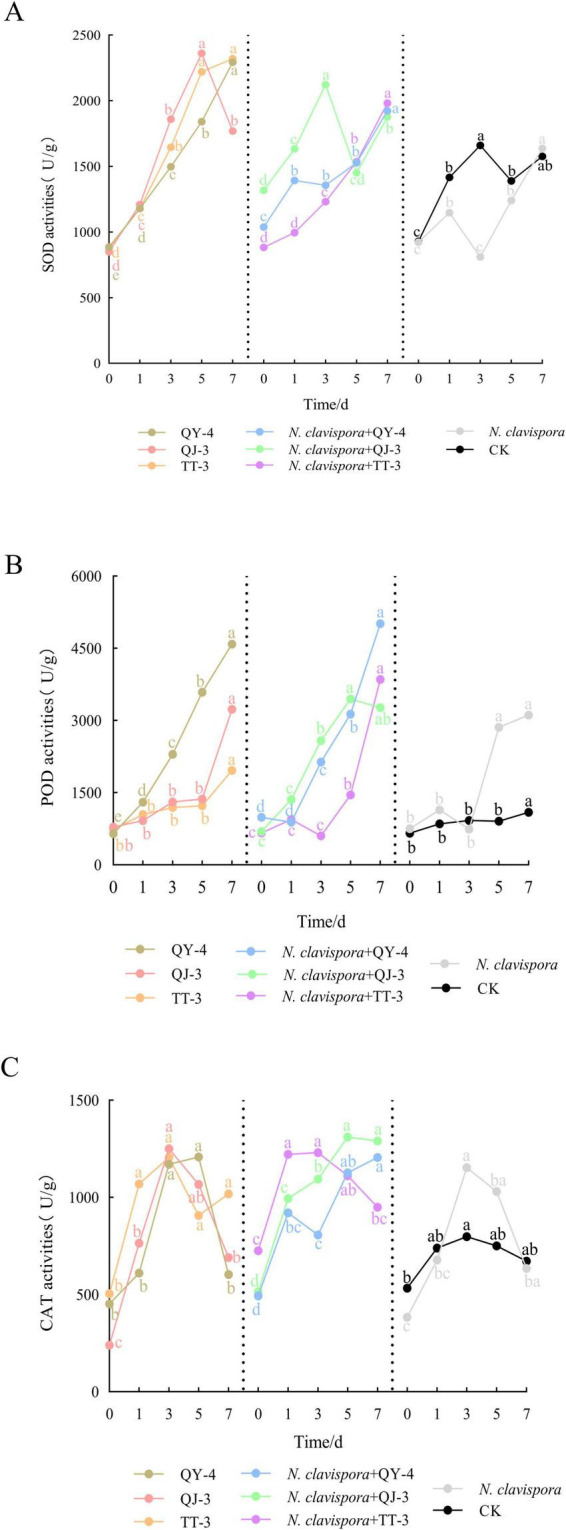
Effects of *N. clavispora* and three antagonistic bacterial strains on defense-related enzymes in strawberry leaves. **(A)** SOD activity in strawberry leaves. **(B)** POD activity in strawberry leaves. **(C)** CAT activity in strawberry leaves. The curves illustrate the effects of eight different treatments on defense enzyme activities in strawberry leaves. The X-axis represents five distinct sampling time points, and the Y-axis indicates enzyme activity. Error bars represent standard deviation (*n* = 3). Different lowercase letters above the bars indicate significant differences in enzyme activity among sampling time points (*p* < 0.05).

Figure 9 illustrates the mechanism by which the three antagonistic strains enhanced strawberry resistance by analyzing the activities of defense-related enzymes in leaves. Across six treatment groups, including QY-4, QJ-3, TT-3, *N. clavispora* + QY-4, *N. clavispora* + QJ-3, and *N. clavispora* + TT-3, the activities of superoxide dismutase (SOD), peroxidase (POD), and catalase (CAT) displayed distinct temporal patterns, with peak levels occurring at different time points.

In all antagonistic treatment groups, the activities of SOD, POD, and CAT were significantly elevated compared with both the *N. clavispora*-infected group and the control (CK), indicating that the antagonistic bacteria induced plant resistance by modulating defense-related enzyme responses. CK exhibited significantly higher SOD activity than the *N. clavispora*-infected group, suggesting that the pathogen suppressed SOD production. In contrast, the *N. clavispora*-infected group showed increased POD and CAT activities compared with CK, reflecting a pathogen-induced oxidative stress response. These results confirmed that strains QY-4, QJ-3, and TT-3 systemically enhanced strawberry resistance by upregulating SOD, POD, and CAT activities, thereby effectively suppressing *N. clavispora* root rot. The consistent induction of defense enzyme responses underscores their potential as biocontrol agents for sustainable disease management.

## 4 Discussion

In recent years, a wide range of antagonistic bacteria has been explored for their potential to control phytopathogenic fungi. These bacteria produce antimicrobial metabolites that inhibit fungal growth by altering cell membrane permeability and disrupting intracellular homeostasis (Salazar et al., 2023). Previous studies have demonstrated the antifungal efficacy of sterile fermented filtrates and volatile organic compounds (VOCs) derived from *Bacillus* species. For instance, sterile filtrates of *Bacillus velezensis* demonstrated inhibition rates of 92.26, 81.82, and 72.73% against *Bipolaris sorokiniana*, *Botrytis cinerea*, and *Colletotrichum capsici*, respectively, while its VOCs exhibited moderate antifungal activity (54, 38, and 22%) against *Fusarium oxysporum*, *Penicillium expansum*, and *Monilinia fructicola* (Huang et al., 2023; Zaid et al., 2023). Similarly, Li Y. et al. (2023) reported that sterile fermented filtrates of *Bacillus subtilis* strain LY-1 significantly suppressed the mycelial growth of *Fusarium oxysporum*, *Fusarium proliferatum*, and *Fusarium solani*, whereas its VOCs exerted a relatively weaker inhibition. Notably, the antifungal activity of sterile filtrates against *Fusarium oxysporum* was positively correlated with their concentration. Consistent with these findings, this study isolated three antagonistic bacterial strains, such as *Bacillus velezensis* strain QY-4, *Bacillus velezensis* strain QJ-3, and *Bacillus subtilis* strain TT-3, from healthy strawberry plants and rhizosphere soil. Sterile filtrates of these strains exhibited strong inhibition against *N. clavispora*, with inhibition rates of 63.29, 69.40, and 73.57%, respectively. Moreover, their antifungal efficacy increased with increasing concentrations, as evidenced by elevated electrolyte leakage and progressive structural damage to fungal hyphae. In contrast, VOCs produced by these strains showed relatively lower inhibition rates (47.76, 44.99, and 32.44%, respectively), suggesting that the non-volatile metabolites in the filtrates served as primary antifungal agents. These results suggested that both the sterile filtrates and VOCs from the three antagonistic strains contributed to the suppression of *N. clavispora*, likely through mechanisms involving disruption of fungal membrane integrity. However, further identification and characterization of the specific bioactive compounds responsible for these effects are necessary.

Biological control strategies involving antagonistic bacteria have emerged as a key research focus for plant disease management. However, current studies have primarily addressed the inhibitory effects of single or limited combinations of strains against individual pathogenic fungi, limiting practical applications owing to narrow antimicrobial spectra, high specificity, and suboptimal efficiency (Li L. et al., 2023). In this study, the three antagonistic strains exhibited not only effective suppression of *N. clavispora* but also broad-spectrum activity against seven other fungal pathogens. Strain QY-4 demonstrated superior antagonistic activity against *Colletotrichum acutatum*, *Alternaria alternata*, and *Botrytis cinerea*, with inhibition rates of 72.59, 72.26, and 74.48%, respectively. Strain QJ-3 markedly inhibited *C. acutatum* (76.54%) and *B. cinerea* (78.88%) and significantly suppressed *A. alternata* (71.09%) and *Botryosphaeria dothidea* (70.12%). Strain TT-3 displayed strong inhibition of *B. cinerea* (76.09%) and moderate activity against *A. alternata* and *C. acutatum*. Collectively, these findings underscore the broad-spectrum antimicrobial potential of the three strains and highlight their applicability as multi-target biocontrol agents in plant protection systems. Their expanded inhibitory spectrum and enhanced efficacy could offer valuable support for the development of consortium-based biocontrol strategies to address the limitations of the current disease management approaches.

In biological control systems, the sustained colonization of antagonistic bacteria within plant tissues and rhizospheric soil constitutes a prerequisite for effective biocontrol functionality. However, the rhizosphere environment presents considerable ecological complexity owing to the coexistence of diverse microbiota, including indigenous rhizospheric microorganisms, phytopathogens, and endophytic flora, which may competitively interfere with the colonization stability of introduced antagonistic strains (Weber and Entrop, 2017). Previous studies have demonstrated that endophytic *Bacillus* species (e.g., *Bacillus subtilis*) exhibit robust colonization capacity in both rhizospheric soil and the plant endosphere, while concurrently enhancing host disease resistance through plant–microbe interactions (Ye et al., 2023; Benizri et al., 2001). Consequently, the ecological competence of antagonistic bacteria to occupy favorable niches within plant tissues and the rhizosphere, coupled with their ability to maintain stable population densities, serves as a critical determinant of their biocontrol potential (Lugtenberg and Kamilova, 2009). In this study, the marked strains of three antagonistic bacteria demonstrated systemic translocation from strawberry roots to stems and leaves, along with persistent colonization in both the plant endosphere and rhizospheric soil. These colonization dynamics indicate their suitability as potential biocontrol agents for plant disease management.

Numerous studies have shown that antagonistic bacteria modulate the activity of defense-related enzymes in host plants, including superoxide dismutase (SOD), peroxidase (POD), and catalase (CAT), thereby enhancing disease resistance. Representative strains such as *Bacillus amyloliquefaciens*, *Bacillus subtilis*, *Bacillus velezensis*, and *Bacillus thuringiensis* have demonstrated this capacity (Achari and Ramesh, 2019; Sahu et al., 2019; Salem et al., 2024). For instance, *Bacillus velezensis* B4-7 significantly elevated SOD, POD, and CAT activities in tobacco leaves, reducing the disease index of bacterial wilt by 74.03% (Meng et al., 2024). Similarly, Chandrasekaran and Chun (2016) reported that tomato plants treated with *Bacillus subtilis* CBR05 fermentation broth exhibited a 36% reduction in soft rot incidence, accompanied by marked increases in SOD, POD, and CAT activities within 48–72 h post-inoculation. In this study, pot experiments revealed that strawberry plants treated with fermentation broths of three antagonistic strains (QY-4, QJ-3, and TT-3) or sterile water exhibited healthy growth. Compared with the *N. clavispora-infected* group, co-inoculation with *N. clavispora* and antagonistic strains (QY-4, QJ-3, and TT-3) resulted in disease index reductions of 36.98, 42.19, and 27.92, respectively, corresponding to biocontrol efficiencies of 56.28, 50.12, and 67%, respectively. These findings confirm the absence of phytotoxicity and highlight the robust efficacy of these strains against strawberry root rot caused by *N. clavispora*. Notably, SOD, POD, and CAT activities in strawberry leaves exhibited dynamic fluctuations across six treatments: QY-4, QJ-3, TT-3, *N. clavispora* + QY-4, *N. clavispora* + QJ-3, and *N. clavispora* + TT-3. The enzymatic activities in all treatment groups were significantly higher than those in the *N. clavispora*-infected and CK groups, indicating that the antagonistic strains induced systemic resistance by modulating defense-related enzyme activities. Intriguingly, SOD activity in the CK group was significantly higher than that in the *N. clavispora*-infected group, whereas the POD and CAT activities showed an inverse trend, suggesting that pathogen infection suppressed SOD activity while transiently activating the POD and CAT responses. Collectively, these results demonstrated that the three antagonistic strains enhanced strawberry resistance to *N. clavispora* by upregulating SOD, POD, and CAT activities. However, further validation through field trials is required to assess the disease control performance and growth impacts under natural agroecological conditions.

The antagonistic strains QY-4, QJ-3, and TT-3 exhibited considerable application potential for the biocontrol of *N. clavispora* root rot in strawberry. It is important to note that the identification of these strains in this study was based on 16S rDNA sequencing. While this method provides a solid preliminary identification, future studies incorporating multi-gene phylogenetic analysis will be essential to achieve a more precise taxonomic resolution. Moreover, the findings of this study provide a scientific basis for developing sustainable management strategies to control this disease effectively. However, to translate this potential into practical application, additional investigations are required to elucidate the specific categories of their antifungal metabolites, clarify their underlying mechanisms of action, and enable systematic validation of their biocontrol effectiveness under field conditions.

## 5 Conclusion

In this study, three high-efficacy, broad-spectrum antagonistic bacterial strains were successfully isolated. These strains were shown to produce antimicrobial metabolites that altered pathogen membrane permeability, exhibited strong colonization capacity in strawberry plants and rhizosphere soil, and enhanced systemic resistance by upregulating SOD, POD, and CAT enzyme activities. As a result, they significantly reduced the disease severity index of strawberry root rot caused by *N. clavispora*, highlighting their substantial potential as sustainable biocontrol agents for managing strawberry root diseases.

## Data Availability

The raw data supporting the conclusions of this article will be made available by the authors, without undue reservation.
