# Supplementary material for: Biocontrol effects of Bacillus velezensis and Bacillus subtilis against strawberry root rot caused by Neopestalotiopsis clavispora
Source: Front Microbiol. 2025 Nov 17;16:1683291. doi: 10.3389/fmicb.2025.1683291 (PMC12667249; doi:10.3389/fmicb.2025.1683291)
Supplement: Supplementary file 1 [file Table_1.docx]

***Supplementary Material***

**Supplementary Table 1.** Antifungal activities of 113 isolated bacteria on mycelial growth of *N. clavispora*

| Strains | Inhibition rate（%） | Strains | Inhibition rate（%） | Strains | Inhibition rate（%） | |
| --- | --- | --- | --- | --- | --- | --- |
| TY-1 | 6.74±0.6 | TG-1 | 6.09±1.15 | TT-8 | 4.6±0.65 | |
| TY-2 | 6.16±0.49 | TG-2 | 4.47±1 | TT-9 | 4.36±0.37 | |
| TY-3 | 4.12±0.72 | TG-3 | 49.04±0.47 | TT-10 | 3.72±0.4 | |
| TY-4 | 3.73±0.44 | TG-4 | 8.3±1.28 | TT-11 | 8.97±0.43 | |
| TY-5 | 2.71±0.04 | TG-5 | 3.44±0.46 | TT-12 | 6.12±1.24 | |
| TY-6 | 3.49±0.64 | TG-6 | 3.6±0.61 | TT-13 | 53.15±0.67 | |
| TY-7 | 7.3±0.58 | TG-7 | 3.43±0.55 | TT-14 | 2.83±0.29 | |
| TY-8 | 5.65±0.35 | TG-8 | 53.45±0.51 | TT-15 | 56.9±2.64 | |
| TJ-1 | 37.91±0.84 | TG-9 | 6.24±0.82 | TT-16 | 6.53±0.51 | |
| TJ-2 | 40.64±0.63 | TG-10 | 9.19±1.09 | TT-17 | 44.45±1.58 | |
| TJ-3 | 36.83±0.81 | TG-11 | 6.35±1.72 | TT-18 | 21.32±1.05 | |
| TJ-4 | 35.53±0.52 | TG-12 | 5.08±0.77 | TT-19 | 50.3±0.51 | |
| TJ-5 | 61.59±0.24 | TG-13 | 36.95±1.06 | TT-20 | 16.1±0.87 | |
| TJ-6 | 38.58±0.28 | TT-1 | 3.49±0.63 | TT-21 | 38.38±1.16 | |
| TJ-7 | 38.42±0.45 | TT-2 | 59.53±1.45 | TT-22 | 37.78±1.3 | |
| TJ-8 | 40.14±1.11 | TT-3 | 60.55±0.3 | QY-1 | 61.14±0.3 | |
| TJ-9 | 51.94±0.46 | TT-4 | 3.46±0.48 | QY-2 | 46.24±1.09 | |
| TJ-10 | 43.92±0.4 | TT-5 | 3.15±0.42 | QY-3 | 35.22±2.41 | |
| TJ-11 | 45.33±0.65 | TT-6 | 4.39±0.81 | QY-4 | 65.12±1.37 | |
| TJ-12 | 54.95±0.43 | TT-7 | 3.21±0.18 | QY-5 | 13.13±0.69 | |
| QY-6 | 14.86±0.84 | QG-8 | 0.47±0.33 | QT-15 | 57.9±1.41 | |
| QY-7 | 1.92±0.65 | QG-9 | 2.55±0.53 | QT-16 | 14.71±4.38 | |
| QJ-1 | 41.19±0.71 | QG-10 | 1.62±0.42 | QT-17 | 9.41±1.15 | |
| QJ-2 | 42.22±0.63 | QG-11 | 4.35±0.74 | QT-18 | 4.43±1.13 | |
| QJ-3 | 65.14±0.49 | QG-12 | 4.69±0.79 | QT-19 | 1.19±0.07 | |
| QJ-4 | 40.52±0.94 | QG-13 | 4.93±0.73 | QT-20 | 3.09±0.55 | |
| QJ-5 | 15.86±1.18 | QG-14 | 4.35±1.09 | QT-21 | 36.64±4.01 | |
| QJ-6 | 49.01±2.14 | QT-1 | 63.38±0.97 | QT-22 | 25.09±0.61 | |
| QJ-7 | 48.73±1.97 | QT-2 | 59.93±0.62 | QT-23 | 6.03±1.69 | |
| QJ-8 | 34.29±0.78 | QT-3 | 59.25±0.59 | QT-24 | 7.57±0.94 | |
| QJ-9 | 0.71±0.51 | QT-4 | 41.1±4.3 | QT-25 | 7.69±0.62 | |
| QJ-10 | 10.26±1.5 | QT-5 | 26.68±0.59 | QG-6 | 52.74±0.72 | |
| QJ-11 | 1.19±0.85 | QT-6 | 26.34±1.23 | QG-7 | 60.74±1.03 | |
| QJ-12 | 43.25±1.64 | QT-7 | 30.13±2.04 | QT-13 | 11.59±1.52 | |
| QG-1 | 40.64±1.86 | QT-8 | 30.39±0.86 | QT-14 | 65.43±0.33 | |
| QG-2 | 7.84±1.53 | QT-9 | 1.9±0.46 | QG-5 | 1.57±0.2 | |
| QG-3 | 3.93±0.33 | QT-10 | 2.95±0.57 | QT-12 | 53.06±0.26 | |
| QG-4 | 5.93±1.37 | QT-11 | 3.08±0.53 |  |  |  |

**Supplementary Table 2.** Physiological and biochemical characteristics of three antagonistic bacterial strains

| Characteristics | | | QY-4 | QJ-3 | TT-3 |
| --- | --- | --- | --- | --- | --- |
| Gram Staining | |  | + | + | + |
| Spore Staining | |  | + | + | + |
| Glucose |  |  | + | + | + |
| Sucrose |  |  | + | + | + |
| Maltose |  |  | + | + | + |
| D-Fructose | |  | + | + | + |
| lactose |  |  | + | + | + |
| Sorbitol |  |  | + | + | + |
| V-P test |  |  | + | + | + |
| Utilization of citrate | |  | + | + | + |
| Methyl red test | |  | - | - | - |
| Nitrate reduction | |  | + | + | + |
| Hydrogen sulfide production | | | + | + | + |
| Indole production | |  | + | + | + |
| Hydrolyzed gelatin | |  | + | + | + |
| Hydrolyzed starch | |  | + | + | + |
| Catalase test | |  | + | + | + |
| Denitrification reaction | | | + | + | + |
| Tyrosine hydrolysis | |  | - | - | - |
| Growth at different pH (5~9) | | | + | + | + |
| Growth at different temperature(20℃~50℃) | | | + | + | + |
| Growth in NaCl at concentration (2% ± 7%) | | | + | + | + |
| Note: “ + ” positive; “-” negative | | |  |  |  |


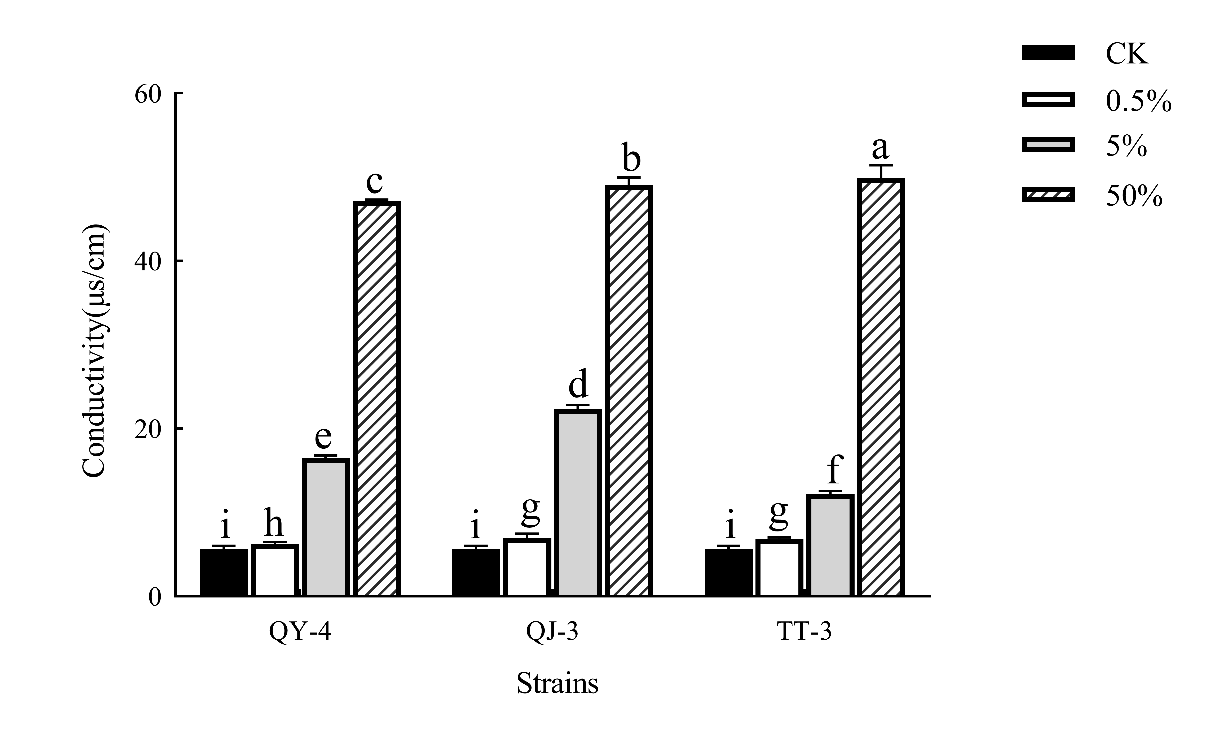


**Supplementary Figure 1.** Effects of different concentrations of sterile fermentation filtrate of three antagonistic bacteria on the cell membrane permeability of *N. clavispora*

Note: X-axis: three antagonistic strains (QY-4, QJ-3, TT-3); Y-axis: electrical conductivity. Error bars: SD (n=3). Different letters indicate significant differences among concentrations (*p*<0.05).
